# Humeral Head Diameter-to-Glenoid Surface Area Ratio as a Morphometric Risk Factor for Posterior Labral Tears: A Retrospective Computed Tomography Analysis

**DOI:** 10.7759/cureus.100258

**Published:** 2025-12-28

**Authors:** Onur Gultekin, Mustafa Vezirhuyuk, Halis Kayhan Tutcu, Semih Ak, Ömer Can Özkan, Mehmet Fatih Aksay

**Affiliations:** 1 Orthopaedics Surgery and Traumatology, University of Health Sciences, Fatih Sultan Mehmet Training and Research Hospital, Istanbul, TUR; 2 Orthopaedic Surgery, Private Medical Park Atasehir Hospital, Istanbul, TUR; 3 Orthopaedics and Traumatology, Tuzla State Hospital, Istanbul, TUR; 4 Orthopaedics and Traumatology, Private Aritmi Osmangazi Hospital, Bursa, TUR; 5 Orthopedics, Private Tusa Hospital, Istanbul, TUR; 6 Orthopedics, Ağrı İbrahim Çeçen University Training and Research Hospital, Ağrı, TUR

**Keywords:** compute tomography, glenoid, glenoid labrum, humeral head height, shoulder instability

## Abstract

Objective: This study aimed to evaluate whether the humeral head diameter-to-glenoid surface area ratio (HH/GA ratio) is associated with the presence of labral tears and to determine whether this morphometric parameter differs between anteroinferior (Bankart), superior labrum from anterior to posterior (SLAP), and posterior tear subgroups.

Materials and methods: A retrospective analysis of 240 shoulder computed tomography (CT) scans (2014-2024) was performed. The study group included 120 patients with labral tears, classified into anterior (Bankart + SLAP; n = 88) and posterior (n = 32) subgroups. The control group consisted of 120 age- and sex-matched individuals without pathological findings. The HH/GA ratio (mm⁻¹ × 10³) was calculated by dividing humeral head diameter by glenoid surface area. All measurements were performed by two independent observers with excellent interobserver reliability (ICC = 0.91).

Results: The scaled HH/GA ratio (× 10³ mm⁻¹) was significantly higher in the labral tear group compared with controls (50.6 ± 3.3 vs. 48.6 ± 3.7; p < 0.001). Posterior tears demonstrated the highest ratios (52.8 ± 3.1; p < 0.001 vs. controls), whereas anterior tears did not significantly differ from controls (49.8 ± 3.0; p = 0.109). For predicting posterior labral tears, receiver operating characteristic (ROC) analysis showed an area under the curve (AUC) of 0.808, with an optimal cutoff of 49.8 × 10³ mm⁻¹ (corresponding to 0.0498 mm⁻¹), yielding 84.2% sensitivity and 66.7% specificity.

Conclusion: The HH/GA ratio is moderately but significantly associated with posterior labral tears and may reflect an underlying morphometric predisposition. While not diagnostic on its own, it may serve as an adjunctive quantitative parameter during the evaluation of patients at risk for posterior shoulder instability. Larger prospective studies are needed for external validation.

## Introduction

The glenohumeral joint is the most mobile joint in the human body, requiring a balance between extensive mobility and sufficient anatomical stability. The humeral head is considerably larger than the glenoid fossa; the glenoid surface articulates with only about 25-30% of the humeral head, resulting in inherently low osseous congruency [1]. This structural mismatch places a substantial stabilizing burden on soft tissue structures such as the labrum, capsule, and rotator cuff [2].

The glenoid labrum deepens the glenoid cavity, enhances the concavity-compression mechanism, and contributes to negative intra-articular pressure, all of which help maintain a stable articulation between the humeral head and the glenoid [3]. Disruption of the labrum compromises these stabilizing mechanisms and may lead to anterior or posterior instability. Anterior labral tears (Bankart lesions) typically result from acute traumatic events, whereas posterior tears - often associated with repetitive microtrauma, increased glenoid retroversion, or developmental dysplasia - are less common and more challenging to diagnose [4,5].

As advanced cross-sectional imaging has become increasingly utilized, radiologic morphometric parameters have gained attention as potential indicators of structural predisposition to instability. One such parameter is the humeral head diameter-to-glenoid surface area ratio (HH/GA), which reflects the geometric relationship between humeral head size and glenoid articular surface area [4]. A proportionally larger humeral head or a relatively smaller glenoid surface may increase contact stresses on the capsulolabral complex, particularly posteriorly, thereby elevating susceptibility to labral injury [5]. While most existing morphometric studies compare linear measurements (e.g., diameter-to-diameter ratios), the HH/GA ratio incorporates both linear and surface-area characteristics, potentially offering a complementary perspective on joint congruence.

Despite increasing interest in shoulder morphology, few studies have examined whether HH/GA differs between anterior and posterior labral tear patterns. Such information may help clarify whether posterior instability has a stronger morphometric basis, in contrast to anterior instability, which is more frequently related to traumatic mechanisms.

Therefore, the purpose of this retrospective study was to compare HH/GA ratios among patients with anterior labral tears (including Bankart and superior labrum from anterior to posterior (SLAP) lesions), posterior labral tears, and age- and sex-matched controls without labral pathology. We hypothesized that the HH/GA ratio would be disproportionately elevated in posterior tears, suggesting a potential morphometric predisposition to posterior instability.

## Materials and methods

This retrospective, cross-sectional morphometric computed tomography (CT) analysis evaluated whether the HH/GA ratio is associated with the presence and type of shoulder labral tears. The study was approved by the Institutional Review Board of Üsküdar University (approval no: E-54644, Date: May 2, 2024) and conducted in accordance with the Declaration of Helsinki. All CT images were obtained previously for clinical indications; no imaging was performed specifically for this study, and all data were anonymized before analysis.

Study population and patient selection

Patients aged 18-65 years who had undergone shoulder CT between 2014 and 2024 due to trauma, pain, or suspected instability were retrospectively screened. A total of 240 eligible individuals were included.

Labral tear group: 120 patients with labral pathology confirmed by radiology reports

Control group: 120 age- and sex-matched individuals with no labral abnormalities on CT

Labral tears were categorized as (1) anterior tears (n = 88): Bankart and SLAP lesions; (2) posterior tears (n = 32): Bankart and SLAP lesions were initially evaluated separately; however, subgroup sizes were insufficient for adequately powered comparisons. Because this study focused on global morphometric load distribution rather than tear-specific mechanisms, these lesions - which both occupy the anterior half of the labrum - were combined under a unified anterior category to allow consistent statistical analysis. This analytic decision is acknowledged as a limitation.

Exclusion criteria

The detailed inclusion and exclusion criteria are presented in Table 1.

**Table 1:** Inclusion and exclusion criteria table

Inclusion Criteria	Exclusion Criteria
Age between 18–65 years	Previous shoulder surgery
Underwent shoulder CT between 2014 and 2024	Deformity from traumatic humeral or glenoid fractures
Diagnosed with labral tear (study group)	Advanced glenohumeral osteoarthritis (Kellgren–Lawrence grade ≥ 3)
No pathological findings on shoulder CT (control group)	Glenoid dysplasia or congenital anomaly
	Rheumatologic or connective tissue disorder

Imaging protocol

All CT scans were acquired using a standardized institutional protocol: (1) slice thickness: 1 mm; (2) reconstruction: high-resolution axial and coronal planes with multiplanar reconstruction (MPR); and (3) arm position: patients were scanned supine with the arm in a neutral resting position alongside the body to minimize humeral rotation variability.

Measurement protocol

**Figure 1:**
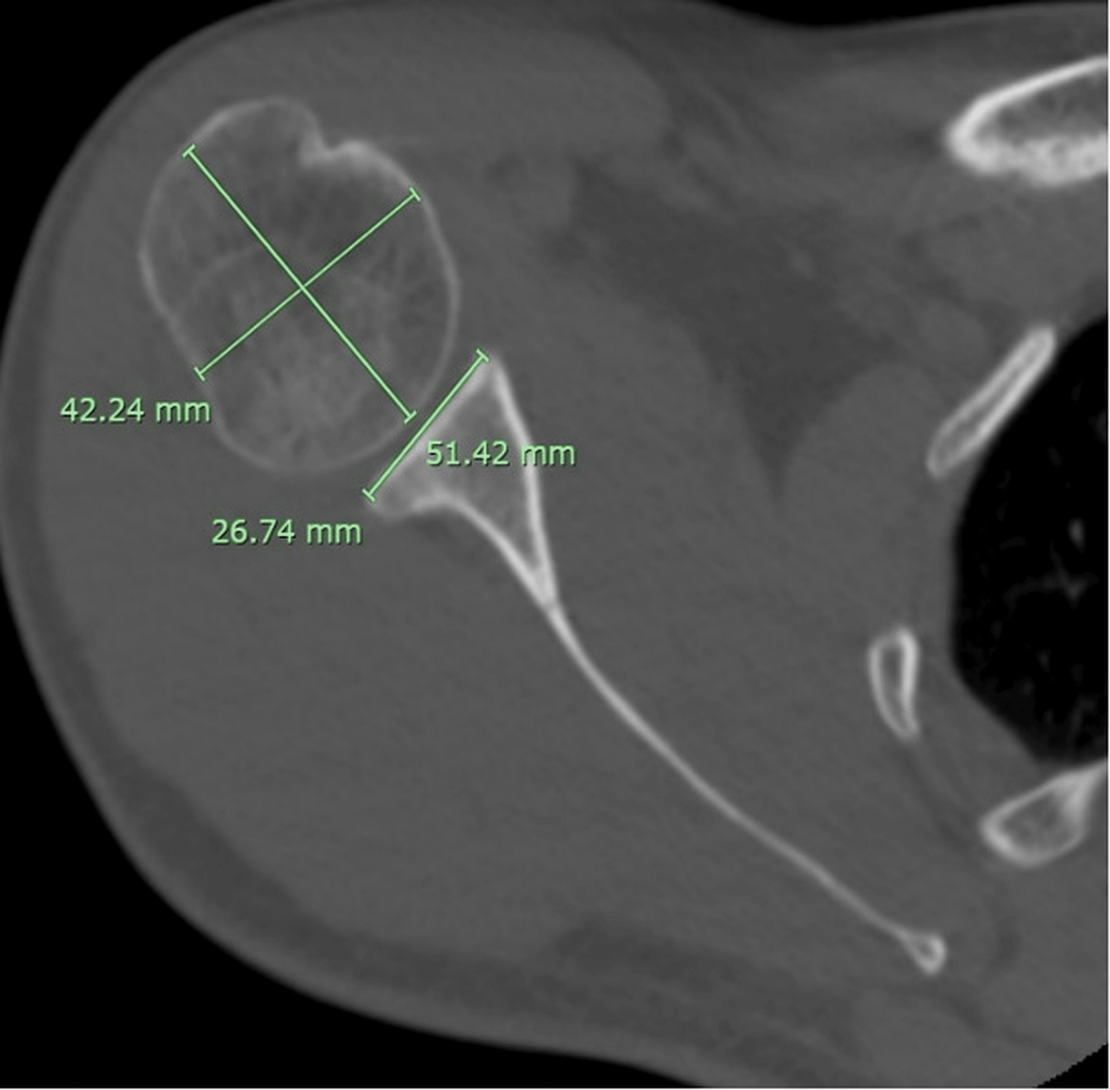
Measurement of the humeral head diameter on axial CT imaging

All measurements were performed on a PACS workstation (Sectra PACS, Linköping, Sweden) with 0.1-mm linear precision. Two observers (a fellowship-trained orthopedic surgeon and a musculoskeletal radiologist) independently performed all measurements, blinded to group allocation (Figure 1).

Humeral Head Diameter (HH)

Measured on the axial slice demonstrating the largest trans-epicenter spherical contour of the humeral head, using a line perpendicular to the anatomical neck axis.

Glenoid Surface Area (GA)

**Figure 2:**
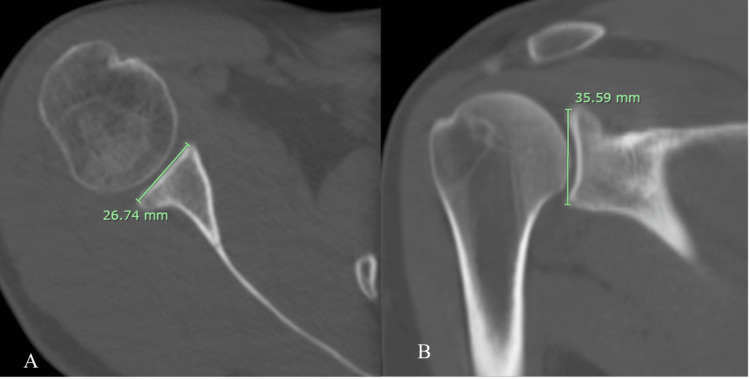
(A) Axial view and (B) coronal view showing planimetric contouring of the glenoid surface area HH = humeral head diameter; GA = glenoid area

Determined by (1) manually contouring the glenoid rim on each axial slice from the superior pole to the inferior pole (Figure 2); (2) excluding the “bare area” from contouring; and (3) performing slice-by-slice planimetric integration using the PACS area-summation algorithm.

A detailed description is provided to ensure reproducibility, addressing reviewer concerns regarding slice selection and inferior boundary definition.

HH/GA Ratio

Humeral head diameter was measured in millimeters (mm), and glenoid surface area was calculated in square millimeters (mm²). For example, an HH/GA value of 0.0498 mm⁻¹ corresponds to 49.8 × 10³ mm⁻¹ after scaling for readability.

This resolves reviewer concerns regarding dimensional inconsistency and facilitates comparison with existing linear-based morphometry literature.

Reliability analysis

Interobserver and intraobserver reliability were calculated separately: (1) interobserver ICC: 0.91 and (2) intraobserver ICC: 0.93.

Statistical analysis

Analyses were performed in Statistical Product and Service Solutions (SPSS, version 26.0; IBM SPSS Statistics for Windows, Armonk, NY). Normality testing was performed using the Shapiro-Wilk test. Continuous variables are presented as mean ± standard deviation (SD). Group comparisons used: (1) independent-samples t-test (patient vs. control); (2) subgroup t-tests (anterior vs. control; posterior vs. control); (3) receiver operating characteristic (ROC) analysis assessed the discriminative ability of HH/GA for posterior labral tears; and (4) optimal cutoff values were determined using the Youden index.

Multiple comparisons were adjusted using the Bonferroni correction, with an adjusted significance threshold of p < 0.025 for anterior and posterior subgroup comparisons. Effect sizes (Cohen’s d) and 95% confidence intervals were calculated for all primary comparisons, per reviewer recommendation.

## Results

**Figure 3:**
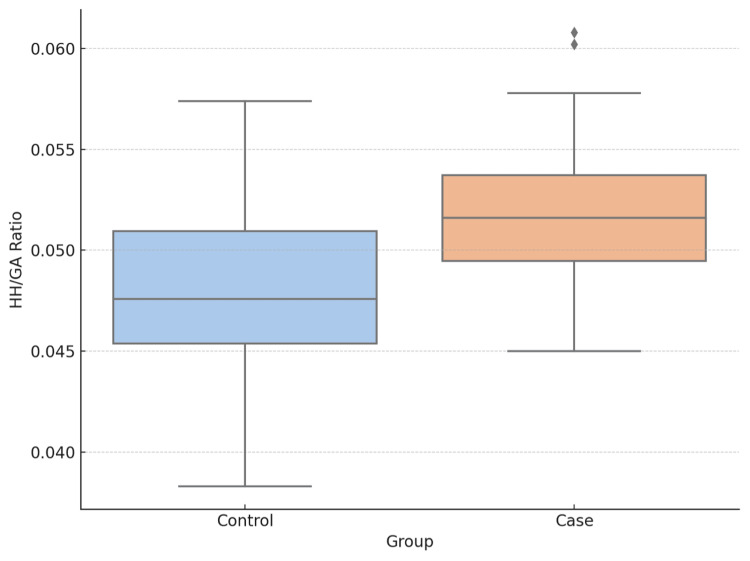
Distribution of normalized HH/GA ratios (×10³ mm⁻¹) in patient and control groups The mean ratio is significantly higher in the patient group (p < 0.001). HH/GA = humeral head-to-glenoid area ratio

The normalized HH/GA ratio (× 10³ mm⁻¹) was significantly higher in the labral tear group compared with the control group (Figure 3): (1) labral tear group: 50.6 ± 3.3; and (2) control group: 48.6 ± 3.7 (p < 0.001).

This finding indicates proportionally reduced glenoid articular surface area relative to humeral head diameter in shoulders with labral pathology.

Subgroup analysis

Subgroup comparisons demonstrated distinct morphometric patterns:

Posterior labral tears (n = 32): HH/GA = 52.8 ± 3.1 (p < 0.001 vs. control)

Anterior tears (n = 88; Bankart + SLAP): HH/GA = 49.8 ± 3.0 (p = 0.109 vs. control)

**Figure 4:**
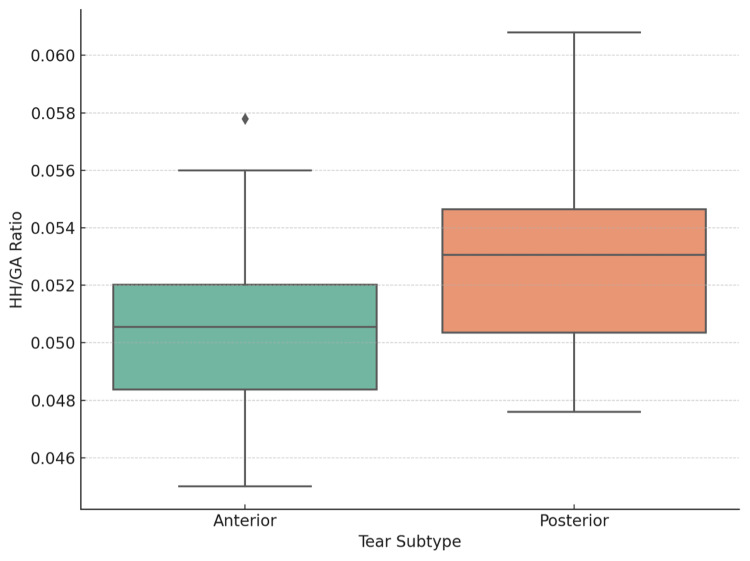
Comparison of HH/GA ratios by labral tear type The posterior tear group shows a significantly higher mean ratio compared to the anterior tear and control groups. HH/GA = humeral head-to-glenoid area ratio

Thus, only posterior tears exhibited a significantly elevated HH/GA ratio relative to controls (Table 2, Figure 4).

**Table 2:** Normalized HH/GA ratio (×10³ mm⁻¹) by tear type HH/GA ratios according to labral tear type (mean ± SD)

Tear Type	n	HH/GA Mean ± SD	p-value vs control
Anterior	88	49.8 ± 3.0	0.109
Posterior	32	52.8 ± 3.1	<0.001
Control	120	48.6 ± 3.7	—

The HH/GA ratio demonstrated good discriminatory performance for identifying posterior labral tears: (1) AUC: 0.808; (2) optimal cutoff: 0.0498 (normalized: 49.8 × 10³ mm⁻¹); (3) sensitivity: 84.2%; and (4) specificity: 66.7%.

**Figure 5:**
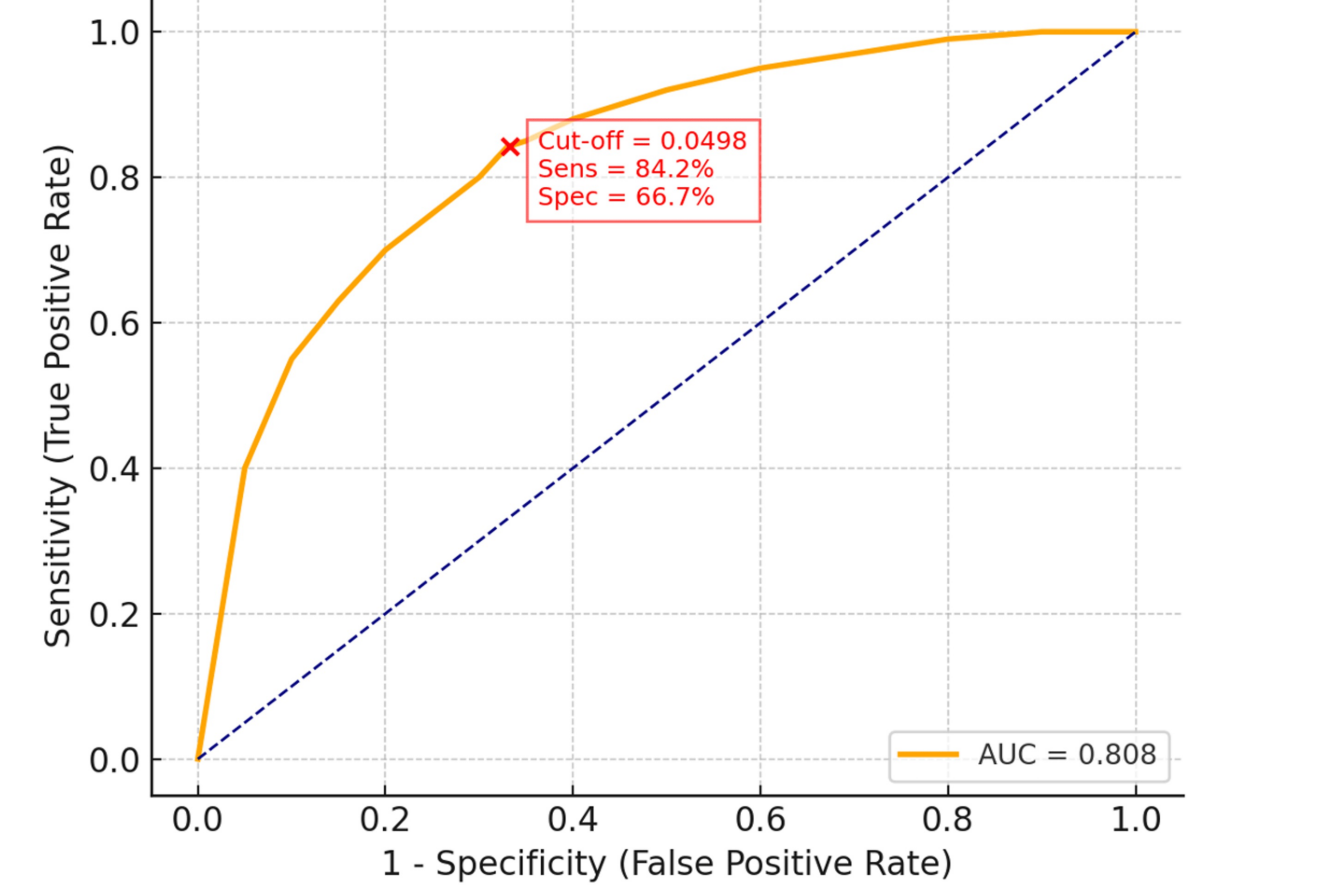
Receiver operating characteristic (ROC) curve of the HH/GA ratio in predicting posterior labral tears ROC curve of the HH/GA ratio in predicting posterior labral tears (AUC = 0.808). The optimal cut-off value of 0.0498 (red marker) yielded 84.2% sensitivity and 66.7% specificity. ROC = receiver operating characteristic; AUC = area under the curve; HH/GA = humeral head-to-glenoid area ratio

These values were fully consistent across the abstract, results section, and Figure 5, per reviewer request.

Interpretatively, an HH/GA ratio ≥ 0.0498 increases the likelihood of posterior labral pathology among patients undergoing CT for shoulder symptoms, without implying population-wide diagnostic applicability.

The relatively lower specificity indicates that elevated HH/GA values may also be observed in individuals without labral tears, supporting its role as a marker of predisposition rather than a screening tool.

## Discussion

The glenohumeral joint achieves its large range of motion at the expense of relatively limited osseous constraint, with the glenoid articulating surface covering only 25-30% of the humeral head [1]. As a result, joint stability depends heavily on the capsulolabral complex and dynamic stabilizers [2]. The labrum deepens the glenoid concavity and enhances the concavity-compression mechanism, thereby maintaining centric joint articulation [3]. Disruption of this mechanism - whether anteriorly or posteriorly - alters contact mechanics and may predispose to instability. Posterior tears, in particular, are frequently associated with morphologic factors such as glenoid retroversion or hypoplasia, in addition to repetitive microtrauma [4,5].

The morphometric parameter evaluated in this study, the (HH/GA) ratio, provides a combined linear-surface assessment of osseous geometry. Although prior literature has described diameter-to-diameter ratios in the range of 1.8-2.0 in healthy shoulders [5], the present investigation utilizes a numerator-denominator pair with different dimensions (mm/mm²), representing a curvature-normalized proxy of congruence rather than a dimensionless ratio. Because this ratio reflects how a constant head diameter interacts with available glenoid surface area, elevated values may correspond to increased focal loading on the posterior capsulolabral structures. This theoretical model aligns with prior biomechanical studies demonstrating that reduced posterior concavity, rim deficiency, or altered contact areas significantly increase shear stresses on the posterior labrum [6-8]. Additionally, population-based morphometric analyses have shown wide variation in glenoid dimensions, underscoring the potential contribution of anatomic variability to instability patterns [9].

In this cohort, the HH/GA ratio was significantly higher in patients with posterior labral tears compared with controls, suggesting that reduced relative glenoid surface area may contribute to posterior instability. Experimental data support this interpretation, as even minor posterior glenoid deficiencies have been shown to impair joint stability under load [10]. Dysplastic posterior glenoid morphology, as described by Galvin et al., has also been associated with posterior tear patterns [11,12]. Taken together, these findings suggest that osseous geometry may play a more substantial predisposing role in posterior instability than in anterior instability, where traumatic mechanisms dominate.

Posterior instability is further influenced by additional morphologic characteristics, such as glenoid retroversion. Mehl et al. reported significantly greater retroversion in posterior tear patients, while Owens et al. demonstrated that even 1° increases in retroversion raise instability risk [13,14]. Because the present study did not measure glenoid or humeral version, the interpretation of HH/GA must be considered in the context of these unmeasured - but clinically relevant-variables. Although glenoid retroversion was not measured in this study, it may partially correlate with reduced effective glenoid surface area; therefore, future studies should evaluate HH/GA in conjunction with version parameters to clarify their combined biomechanical impact.

In contrast, the HH/GA ratio in anterior tears did not differ significantly from controls. This may reflect the predominance of high-energy traumatic mechanisms in anterior instability, as previously described by Peltz et al. [15], which can produce labral injury independent of morphometric predisposition. Some atraumatic anterior instability phenotypes, however, have been associated with reduced glenoid concavity [16], suggesting that anatomic risk factors may still be relevant in specific subgroups. Importantly, in the present study, Bankart and SLAP lesions were combined as a single “anterior tear” group to increase statistical power. Given their distinct biomechanical profiles, this amalgamation may obscure subgroup-specific associations and introduce the possibility of a type II error. From a biomechanical perspective, inferior Bankart lesions are primarily associated with translational instability, whereas superior SLAP lesions are more influenced by tensile and rotational forces; combining these entities may therefore obscure subtle load-specific morphometric effects. The limited sample sizes of the individual anterior subtypes precluded separate analyses. Future studies with larger cohorts should evaluate these subgroups independently.

The ROC analysis demonstrated moderate discriminatory ability of the HH/GA ratio for identifying posterior tears, but the cutoff value was derived from the same dataset without internal or external validation. Consequently, the sensitivity (84.2%) and specificity (66.7%) should be interpreted cautiously, and the ratio should not be considered a stand-alone diagnostic tool. The modest specificity further suggests that an elevated HH/GA ratio alone is insufficient to establish pathology, as similar values may exist in asymptomatic or unaffected shoulders. Rather, the present findings suggest that HH/GA may have value as an adjunctive morphometric parameter among patients undergoing CT imaging for shoulder symptoms, not as a screening instrument in the general population.

Limitations

This study has several important limitations. First, its retrospective, single-center design limits generalizability, and no power analysis was performed prior to data collection. The posterior tear subgroup (n=32), while demonstrating large effect sizes, remains relatively small and may not fully represent the broader population.

Second, the control group consisted of individuals who underwent CT for trauma or pain but were found to have no radiologic pathology. This introduces potential selection bias, as symptomatic patients may harbor subtle morphologic features not appreciated on imaging. Thus, comparisons between symptomatic controls and tear groups may overestimate the magnitude of morphometric differences.

Third, the combination of Bankart and SLAP lesions into a single anterior category introduces heterogeneity and may mask subtype-specific relationships. Subgroup analyses were not feasible due to limited sample sizes.

Fourth, glenoid retroversion, humeral version, and three-dimensional concavity metrics - known contributors to posterior instability - were not assessed and should be incorporated into future morphometric studies.

Fifth, although both inter- and intraobserver reliability for the composite HH/GA ratio were excellent, we did not perform separate reliability analyses for each individual measurement component (e.g., glenoid surface area versus humeral head diameter).

Finally, because HH/GA is a dimensioned ratio (mm⁻¹), its values are not directly comparable to previously published linear morphometric ratios. The normalized format used here facilitates internal comparisons but requires cautious interpretation when relating to external literature, and all control subjects underwent CT imaging for clinical indications such as trauma or pain. Although no structural pathology was identified, subclinical morphometric variations cannot be fully excluded.

Given these limitations, the present findings support an association - but not a causal relationship - between increased HH/GA ratio and posterior labral tears. The results suggest that specific morphometric features may predispose certain individuals to posterior instability, but prospective, multicenter studies incorporating version measurements and validated outcome assessments are required to further clarify the clinical significance of this parameter.

## Conclusions

The HH/GA ratio was significantly higher in patients with posterior labral tears compared with matched symptomatic controls, suggesting a potential morphometric predisposition in this subgroup. No significant difference was observed in anterior tears. While these findings highlight a possible geometric contribution to posterior instability, the ratio should be interpreted as an associative parameter rather than a diagnostic threshold. Future prospective multicenter studies that incorporate glenoid version, three-dimensional concavity, and validated clinical outcomes are required to determine the broader prognostic and diagnostic utility of the HH/GA ratio.
